# Clinical utility of repeated blood culture sampling in critically ILL neonates

**DOI:** 10.1186/2197-425X-3-S1-A556

**Published:** 2015-10-01

**Authors:** EH Verstraete, L Mahieu, JD' Haese, K De Coen, J Boelens, D Vogelaers, S Blot

**Affiliations:** Internal Medicine, Ghent University, Ghent, Belgium; Pediatrics, University of Antwerp, Antwerp, Belgium; Neonatal Medicine, Antwerp University Hospital, Antwerp, Belgium; Neonatal Medicine, General Hospital Sint-Jan, Bruges, Belgium; Neonatal Medicine, Ghent University Hospital, Ghent, Belgium; Laboratory Medicine, Ghent University Hospital, Ghent, Belgium; General Internal Medicine, Ghent University Hospital, Ghent, Belgium

## Introduction

Repeated blood culture sampling is common in critically ill neonates though precise indications are unknown.

## Objectives

To describe clinical characteristics driving repeated blood culture sampling: clinical indications, time interval between cultures, and rates of blood culture-positivity.

## Methods

Prospective multicenter study of characteristics for repeated blood culture sampling in neonates admitted to three tertiary-referral intensive care units (period 7/2013-12/2014). Repeated blood culture samples were included when obtained within 14 days after the previous sample. Clinical sepsis is defined as the presence of 2 clinical signs and the duration of antibiotic therapy for ≥5 days. Blood cultures positive for skin commensals are considered contaminated if no 2 clinical signs and no CRP of > 2mg/dL are identified.

## Results

**Figure 1:**
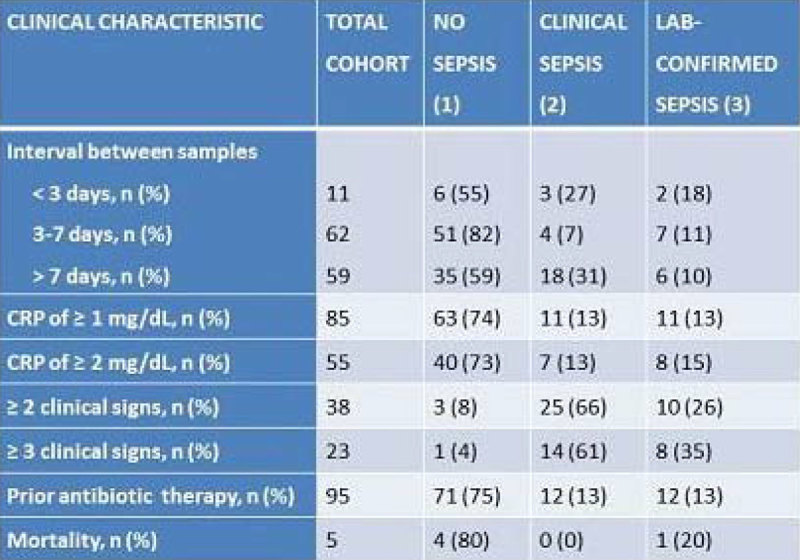
**Characteristics of 132 included cultures.**

Of the 413 initial blood culture samples in 286 neonates, 132 (32%) were repeated blood cultures sampled in 97 neonates, 42 of which had a birth-weight ≤1500 g. Repeated cultures resulted in: (1) no sepsis, i.e. no growth (n = 87, 65.9%) and contamination (n = 5, 3.8%), (2) clinical sepsis (n = 25, 18.9%), and (3) lab-confirmed sepsis (n = 15, 11.4%). Clinical characteristics of repeated cultures for the total and those three cohorts are in Figure 1. Significant less clinical signs were observed between the cohort of neonates receiving prior antibiotic therapy (ABT, n = 95) vs no prior ABT (n = 37) (median 0 [IQR 0-1] vs median 1 [IQR 0-3], *P* =. 016); also shorter interval between cultures (median 5 [IQR 3-8] vs median 11 [IQR 9-13], *P* < .001) and higher CRP values (median 2 [IQR 1-4] vs median 1 [IQR 0-2], *P* < .001) were noticed. No significant difference in lab-confirmed sepsis was observed between the prior ABT vs no prior ABT cohort.

## Conclusions

CRP rise seems not a good indicator for repeated blood culturing, though mostly identified as an indicator in particular in the prior-ABT-cohort. Prior ABT influences indications and interval for repeated cultures but has no effect on blood culture-positivity. Repeated blood culture samples seems indicated when ≥2 clinical signs occur.

## Grant Acknowledgment

Grant by Belgian Research Fund (BOF)

## References

[1] Al-Lawama MA, Badran EF (2015). Clinical value of repeat blood cultures in neonatal patients receiving antibiotic treatment. J Int Med Res.

[2] Tabriz MS, Riederer K, Baran J, Khatib R (2004). Repeating blood cultures during hospital stay: practice pattern at a teaching hospital and a proposal for guidelines. Clin Microbiol Infect.

